# 6,6′-Dimethyl-2,2′-[imidazolidine-1,3-diyl­bis(methyl­ene)]diphenol

**DOI:** 10.1107/S1600536814002128

**Published:** 2014-02-05

**Authors:** Augusto Rivera, Luz Stella Nerio, Michael Bolte

**Affiliations:** a Departamento de Química, Facultad de Ciencias, Universidad Nacional de Colombia, Sede Bogotá, Cra 30 No. 45-03, Bogotá, Colombia; b Institut für Anorganische Chemie, J. W. Goethe-Universität Frankfurt, Max-von-Laue-Strasse 7, 60438 Frankfurt/Main, Germany

## Abstract

In the title compound, C_19_H_24_N_2_O_2_, a di-Mannich base derived from 2-methyl­phenol and 1,3,6,8-tetra­aza­tri­cyclo­[4.4.1.1^3,8^]dodecane, the imidazolidine ring adopts a twist conformation, with a twist about the ring N—C bond [C—N—C—C torsion angle = −44.34 (14)°]. The two 2-hy­droxy-3-methyl­benzyl groups are located in *trans* positions with respect to the imidazolidine fragment. The structure displays two intra­molecular O—H⋯N hydrogen bonds, which each form an *S*(6) ring motif. In the crystal, the mol­ecules are linked by weak C—H⋯O inter­actions with a bifurcated acceptor, forming a three-dimensional network.

## Related literature   

For the original synthesis of the title compound, see: Rivera *et al.* (1993 ▶). For related structures, see: Rivera *et al.* (2011 ▶, 2012*a* ▶,*b* ▶,*c* ▶, 2013 ▶). For hydrogen-bond motifs, see: Bernstein *et al.* (1995 ▶). For ring conformations, see Cremer & Pople (1975 ▶). For bifurcated-acceptor hydrogen-bond conformations, see: Desiraju & Steiner (1999 ▶).
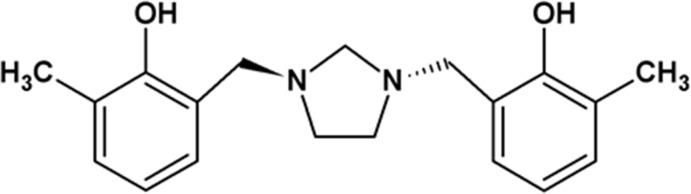


## Experimental   

### 

#### Crystal data  

C_19_H_24_N_2_O_2_
*M* *_r_* = 312.40
Monoclinic, 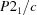

*a* = 12.6271 (11) Å
*b* = 13.5780 (9) Å
*c* = 10.1997 (9) Å
β = 107.940 (7)°
*V* = 1663.7 (2) Å^3^
*Z* = 4
Mo *K*α radiation
μ = 0.08 mm^−1^
*T* = 173 K
0.33 × 0.13 × 0.12 mm

#### Data collection  

Stoe IPDS II two-circle diffractometer
Absorption correction: multi-scan (*X-AREA*; Stoe & Cie, 2001 ▶) *T*_min_ = 0.974, *T*_max_ = 0.990
16285 measured reflections
3207 independent reflections
2803 reflections with *I* > 2σ(*I*)
*R*_int_ = 0.072

#### Refinement  

*R*[*F* ^2^ > 2σ(*F* ^2^)] = 0.043
*wR*(*F* ^2^) = 0.114
*S* = 1.07
3207 reflections
219 parameters
H atoms treated by a mixture of independent and constrained refinement
Δρ_max_ = 0.20 e Å^−3^
Δρ_min_ = −0.17 e Å^−3^

### 

Data collection: *X-AREA* (Stoe & Cie, 2001 ▶); cell refinement: *X-AREA*; data reduction: *X-AREA*; program(s) used to solve structure: *SHELXS97* (Sheldrick, 2008 ▶); program(s) used to refine structure: *SHELXL97* (Sheldrick, 2008 ▶); molecular graphics: *XP* in *SHELXTL-Plus* (Sheldrick, 2008 ▶); software used to prepare material for publication: *SHELXL97*.

## Supplementary Material

Crystal structure: contains datablock(s) I, New_Global_Publ_Block. DOI: 10.1107/S1600536814002128/fb2295sup1.cif

Structure factors: contains datablock(s) I. DOI: 10.1107/S1600536814002128/fb2295Isup2.hkl

Supporting information file. DOI: 10.1107/S1600536814002128/fb2295Isup3.cml

CCDC reference:

Additional supporting information:  crystallographic information; 3D view; checkCIF report

## Figures and Tables

**Table 1:** Hydrogen-bond geometry (Å, °)

*D*—H⋯*A*	*D*—H	H⋯*A*	*D*⋯*A*	*D*—H⋯*A*
O1—H1⋯N1	0.91 (2)	1.90 (2)	2.7115 (16)	146 (2)
O2—H2⋯N2	0.98 (2)	1.70 (2)	2.6202 (16)	155 (2)
C5—H5*B*⋯O2^i^	0.99	2.43	3.4012 (18)	167
C27—H27*A*⋯O2^ii^	0.98	2.50	3.1789 (19)	126

Symmetry codes: (i) 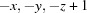; (ii) 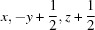.

**Fig. 1.**
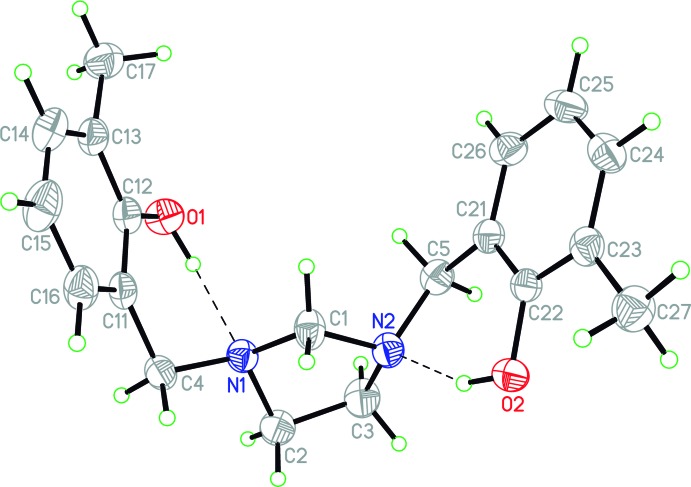
The title molecule with the atom-numbering scheme·The displacement ellipsoids are shown at the 50% probability level. The hydrogen bonds are drawn as dashed lines.

## supplementary crystallographic information

### 1. Comment

The title molecule with the atom-numbering scheme is shown in Fig 1. The 2-hydroxy-3-methylbenzyl substituents are arranged in *trans* configuration with respect to the imidazolidine ring. The imidazolidine ring adopts a twist conformation, with a C1—N1—C2—C3 torsion angle equal to -44.34 (14)° (Cremer & Pople, 1975).

The structure of the title compound shows the presence of two intramolecular O—H···N hydrogen bonds. These bond take part in the S(6) graph set motifs (Bernstein *et al.*, 1995). These motifs occur in the related 2,2'-[imidazolidine-1,3-diylbis(methylene)]diphenol compounds (Rivera *et al.* 2011, 2012*a*, *b*, *c*, 2013), too. The two intramolecular hydrogen bonds are different in their length (Table 1), although both nitrogen atoms are connected to the same atomic species. In the crystal, the molecules are linked to each other by weak C—H···O interactions (Desiraju & Steiner, 1999) C5—H5B···O2^i^···H27A^iii^—C27^iii^ with a bifurcated acceptor O2^i^ where (i): -*x*, -*y*, -*z*+1; (iii): -*x*, *y*-1/2, -*z*+3/2. (For the hydrogen bonds and interactions, see Table 1.)

### 2. Experimental

For the original synthesis of the title compound, see Rivera *et al.* (1993). Single crystals in the form of needles that were shorter than 1 mm were obtained by slow evaporation of 0.01 *M* ethanol solution at room temperature. Melting point: 403-404 K.

### 3. Refinement

All the H atoms were located in the difference electron density map. The hydroxyl H atoms were refined freely, however, the H atoms bonded to C atoms were situated into the idealized positions and refined using a riding model approximation. The applied constraints were as follows: C_methylene_—H = 0.99Å, C_methyl_—H = 0.98Å and C_aryl_—H = 0.95Å. *U*_iso_(H_aryl/methylene_) = 1.2*U*_eq_(C_aryl/methylene_) except for *U*_iso_(H_methyl_) = 1.5*U*_eq_(C_methyl_). The methyl groups were allowed to rotate during the refinement by application of the command AFIX 137 (*SHELXL97* (Sheldrick, 2008)).

### Crystal data

**Table:**

C_19_H_24_N_2_O_2_	*D*_x_ = 1.247 Mg m^−^^3^
*M**_r_* = 312.40	Melting point = 403–404 K
Monoclinic, *P*2_1_/*c*	Mo *K*α radiation, λ = 0.71073 Å
*a* = 12.6271 (11) Å	Cell parameters from 17856 reflections
*b* = 13.5780 (9) Å	θ = 3.4–26.3°
*c* = 10.1997 (9) Å	µ = 0.08 mm^−^^1^
β = 107.940 (7)°	*T* = 173 K
*V* = 1663.7 (2) Å^3^	Needle, brown
*Z* = 4	0.33 × 0.13 × 0.12 mm
*F*(000) = 672	

### Data collection

**Table:**

Stoe IPDS II two-circle diffractometer	3207 independent reflections
Radiation source: Genix 3D IµS microfocus X-ray source	2803 reflections with *I* > 2σ(*I*)
Genix 3D multilayer optics monochromator	*R*_int_ = 0.072
ω scans	θ_max_ = 25.9°, θ_min_ = 3.4°
Absorption correction: multi-scan (*X-AREA*; Stoe & Cie, 2001)	*h* = −15→15
*T*_min_ = 0.974, *T*_max_ = 0.990	*k* = −16→14
16285 measured reflections	*l* = −12→12

### Refinement

**Table:**

Refinement on *F*^2^	Secondary atom site location: difference Fourier map
Least-squares matrix: full	Hydrogen site location: difference Fourier map
*R*[*F*^2^ > 2σ(*F*^2^)] = 0.043	H atoms treated by a mixture of independent and constrained refinement
*wR*(*F*^2^) = 0.114	*w* = 1/[σ^2^(*F*_o_^2^) + (0.053*P*)^2^ + 0.4801*P*] where*P* = (*F*_o_^2^ + 2*F*_c_^2^)/3
*S* = 1.07	(Δ/σ)_max_ < 0.001
3207 reflections	Δρ_max_ = 0.20 e Å^−^^3^
219 parameters	Δρ_min_ = −0.17 e Å^−^^3^
0 restraints	Extinction correction: *SHELXL97* (Sheldrick, 2008), Fc^*^=kFc[1+0.001xFc^2^λ^3^/sin(2θ)]^-1/4^
Primary atom site location: difference Fourier map	Extinction coefficient: 0.017 (3)

### Special details

**Table:**

Geometry. All e.s.d.'s (except the e.s.d. in the dihedral angle between two l.s. planes) are estimated using the full covariance matrix. The cell e.s.d.'s are taken into account individually in the estimation of e.s.d.'s in distances, angles and torsion angles; correlations between e.s.d.'s in cell parameters are only used when they are defined by crystal symmetry. An approximate (isotropic) treatment of cell e.s.d.'s is used for estimating e.s.d.'s involving l.s. planes.
Refinement. Refinement of *F*^2^ against ALL reflections. The weighted *R*-factor *wR* and goodness of fit *S* are based on *F*^2^, conventional *R*-factors *R* are based on *F*, with *F* set to zero for negative *F*^2^. The threshold expression of *F*^2^ > σ(*F*^2^) is used only for calculating *R*-factors(gt) *etc*. and is not relevant to the choice of reflections for refinement. *R*-factors based on *F*^2^ are statistically about twice as large as those based on *F*, and *R*-factors based on ALL data will be even larger.

### Fractional atomic coordinates and isotropic or equivalent isotropic displacement parameters (Å^2^)

**Table:**

	*x*	*y*	*z*	*U*_iso_*/*U*_eq_	
O1	0.52432 (9)	0.10588 (7)	0.59144 (11)	0.0354 (3)	
H1	0.460 (2)	0.1024 (17)	0.519 (2)	0.067 (7)*	
O2	−0.00181 (8)	0.17557 (8)	0.53718 (10)	0.0326 (3)	
H2	0.047 (2)	0.1533 (18)	0.485 (2)	0.070 (7)*	
N1	0.32247 (9)	0.16347 (9)	0.42350 (11)	0.0268 (3)	
N2	0.16437 (9)	0.10080 (9)	0.46722 (12)	0.0290 (3)	
C1	0.25284 (12)	0.17521 (11)	0.51349 (15)	0.0317 (3)	
H1A	0.2207	0.2423	0.5046	0.038*	
H1B	0.2969	0.1639	0.6109	0.038*	
C2	0.24302 (12)	0.13405 (12)	0.29153 (14)	0.0344 (3)	
H2A	0.2812	0.1047	0.2295	0.041*	
H2B	0.1970	0.1904	0.2447	0.041*	
C3	0.17414 (13)	0.05850 (13)	0.33757 (15)	0.0382 (4)	
H3A	0.1000	0.0505	0.2682	0.046*	
H3B	0.2122	−0.0062	0.3542	0.046*	
C4	0.38508 (12)	0.25297 (11)	0.41628 (15)	0.0306 (3)	
H4A	0.3329	0.3089	0.3864	0.037*	
H4B	0.4247	0.2440	0.3471	0.037*	
C5	0.16693 (12)	0.02503 (11)	0.57129 (15)	0.0314 (3)	
H5A	0.2444	0.0013	0.6112	0.038*	
H5B	0.1208	−0.0316	0.5255	0.038*	
C11	0.46819 (11)	0.27610 (10)	0.55457 (15)	0.0282 (3)	
C12	0.53386 (11)	0.20167 (10)	0.63485 (15)	0.0278 (3)	
C13	0.61017 (11)	0.22221 (11)	0.76376 (15)	0.0311 (3)	
C14	0.62035 (12)	0.31874 (12)	0.80964 (17)	0.0381 (4)	
H14	0.6729	0.3341	0.8962	0.046*	
C15	0.55590 (13)	0.39346 (12)	0.73245 (19)	0.0424 (4)	
H15	0.5636	0.4591	0.7662	0.051*	
C16	0.48010 (12)	0.37156 (11)	0.60557 (17)	0.0356 (3)	
H16	0.4356	0.4227	0.5526	0.043*	
C17	0.67528 (13)	0.14002 (13)	0.85113 (17)	0.0411 (4)	
H17A	0.7204	0.1664	0.9402	0.062*	
H17B	0.7240	0.1097	0.8042	0.062*	
H17C	0.6237	0.0904	0.8659	0.062*	
C21	0.12514 (11)	0.06119 (10)	0.68633 (14)	0.0272 (3)	
C22	0.04162 (11)	0.13237 (10)	0.66313 (13)	0.0261 (3)	
C23	−0.00336 (12)	0.16134 (11)	0.76666 (15)	0.0296 (3)	
C24	0.03670 (13)	0.11608 (11)	0.89454 (15)	0.0335 (3)	
H24	0.0066	0.1339	0.9658	0.040*	
C25	0.11964 (14)	0.04555 (11)	0.92013 (15)	0.0368 (4)	
H25	0.1461	0.0155	1.0084	0.044*	
C26	0.16402 (12)	0.01877 (11)	0.81681 (15)	0.0330 (3)	
H26	0.2216	−0.0291	0.8352	0.040*	
C27	−0.09425 (13)	0.23731 (12)	0.73391 (17)	0.0391 (4)	
H27A	−0.1177	0.2491	0.8156	0.059*	
H27B	−0.1578	0.2136	0.6584	0.059*	
H27C	−0.0665	0.2988	0.7063	0.059*	

### Atomic displacement parameters (Å^2^)

**Table:**

	*U*^11^	*U*^22^	*U*^33^	*U*^12^	*U*^13^	*U*^23^
O1	0.0403 (6)	0.0253 (5)	0.0376 (6)	0.0031 (4)	0.0076 (5)	−0.0018 (4)
O2	0.0313 (5)	0.0376 (6)	0.0286 (5)	0.0036 (4)	0.0090 (4)	0.0072 (4)
N1	0.0281 (6)	0.0290 (6)	0.0252 (6)	−0.0013 (5)	0.0109 (5)	−0.0026 (4)
N2	0.0299 (6)	0.0310 (6)	0.0269 (6)	−0.0058 (5)	0.0097 (5)	−0.0056 (5)
C1	0.0318 (7)	0.0343 (8)	0.0332 (7)	−0.0078 (6)	0.0163 (6)	−0.0096 (6)
C2	0.0354 (7)	0.0427 (9)	0.0262 (7)	−0.0025 (6)	0.0111 (6)	−0.0060 (6)
C3	0.0414 (8)	0.0431 (9)	0.0312 (7)	−0.0104 (7)	0.0128 (6)	−0.0131 (6)
C4	0.0300 (7)	0.0311 (7)	0.0329 (7)	0.0001 (6)	0.0130 (6)	0.0059 (6)
C5	0.0308 (7)	0.0277 (7)	0.0350 (8)	−0.0012 (6)	0.0090 (6)	−0.0020 (6)
C11	0.0241 (6)	0.0285 (7)	0.0354 (7)	−0.0028 (5)	0.0141 (6)	0.0021 (6)
C12	0.0257 (6)	0.0264 (7)	0.0345 (7)	−0.0014 (5)	0.0142 (6)	0.0003 (6)
C13	0.0252 (7)	0.0340 (8)	0.0361 (7)	−0.0024 (6)	0.0126 (6)	0.0007 (6)
C14	0.0295 (7)	0.0380 (9)	0.0443 (9)	−0.0083 (6)	0.0076 (6)	−0.0062 (7)
C15	0.0374 (8)	0.0278 (8)	0.0600 (10)	−0.0073 (6)	0.0119 (7)	−0.0095 (7)
C16	0.0311 (7)	0.0254 (7)	0.0507 (9)	−0.0015 (6)	0.0130 (6)	0.0026 (6)
C17	0.0380 (8)	0.0424 (9)	0.0385 (8)	0.0014 (7)	0.0055 (7)	0.0039 (7)
C21	0.0269 (6)	0.0237 (7)	0.0297 (7)	−0.0060 (5)	0.0068 (5)	−0.0015 (5)
C22	0.0260 (6)	0.0247 (7)	0.0261 (6)	−0.0073 (5)	0.0057 (5)	0.0007 (5)
C23	0.0292 (7)	0.0281 (7)	0.0316 (7)	−0.0076 (5)	0.0095 (6)	−0.0041 (5)
C24	0.0418 (8)	0.0325 (8)	0.0280 (7)	−0.0102 (6)	0.0137 (6)	−0.0047 (6)
C25	0.0471 (9)	0.0335 (8)	0.0259 (7)	−0.0064 (7)	0.0053 (6)	0.0045 (6)
C26	0.0333 (7)	0.0275 (7)	0.0333 (7)	−0.0018 (6)	0.0031 (6)	0.0028 (6)
C27	0.0373 (8)	0.0414 (9)	0.0400 (8)	0.0013 (7)	0.0140 (7)	−0.0039 (7)

### Geometric parameters (Å, º)

**Table:**

O1—C12	1.3673 (17)	C12—C13	1.398 (2)
O1—H1	0.91 (2)	C13—C14	1.384 (2)
O2—C22	1.3643 (16)	C13—C17	1.505 (2)
O2—H2	0.98 (2)	C14—C15	1.384 (2)
N1—C1	1.4622 (17)	C14—H14	0.9500
N1—C4	1.4639 (18)	C15—C16	1.384 (2)
N1—C2	1.4650 (18)	C15—H15	0.9500
N2—C5	1.4714 (19)	C16—H16	0.9500
N2—C1	1.4719 (17)	C17—H17A	0.9800
N2—C3	1.4813 (18)	C17—H17B	0.9800
C1—H1A	0.9900	C17—H17C	0.9800
C1—H1B	0.9900	C21—C26	1.393 (2)
C2—C3	1.510 (2)	C21—C22	1.396 (2)
C2—H2A	0.9900	C22—C23	1.401 (2)
C2—H2B	0.9900	C23—C24	1.389 (2)
C3—H3A	0.9900	C23—C27	1.502 (2)
C3—H3B	0.9900	C24—C25	1.383 (2)
C4—C11	1.509 (2)	C24—H24	0.9500
C4—H4A	0.9900	C25—C26	1.386 (2)
C4—H4B	0.9900	C25—H25	0.9500
C5—C21	1.509 (2)	C26—H26	0.9500
C5—H5A	0.9900	C27—H27A	0.9800
C5—H5B	0.9900	C27—H27B	0.9800
C11—C16	1.388 (2)	C27—H27C	0.9800
C11—C12	1.400 (2)		
			
C12—O1—H1	106.2 (15)	C13—C12—C11	121.23 (13)
C22—O2—H2	103.8 (14)	C14—C13—C12	118.17 (14)
C1—N1—C4	112.31 (11)	C14—C13—C17	121.68 (14)
C1—N1—C2	103.44 (11)	C12—C13—C17	120.11 (14)
C4—N1—C2	114.07 (11)	C13—C14—C15	121.64 (15)
C5—N2—C1	113.75 (11)	C13—C14—H14	119.2
C5—N2—C3	112.55 (12)	C15—C14—H14	119.2
C1—N2—C3	106.78 (11)	C16—C15—C14	119.36 (15)
N1—C1—N2	105.53 (11)	C16—C15—H15	120.3
N1—C1—H1A	110.6	C14—C15—H15	120.3
N2—C1—H1A	110.6	C15—C16—C11	121.00 (14)
N1—C1—H1B	110.6	C15—C16—H16	119.5
N2—C1—H1B	110.6	C11—C16—H16	119.5
H1A—C1—H1B	108.8	C13—C17—H17A	109.5
N1—C2—C3	101.34 (11)	C13—C17—H17B	109.5
N1—C2—H2A	111.5	H17A—C17—H17B	109.5
C3—C2—H2A	111.5	C13—C17—H17C	109.5
N1—C2—H2B	111.5	H17A—C17—H17C	109.5
C3—C2—H2B	111.5	H17B—C17—H17C	109.5
H2A—C2—H2B	109.3	C26—C21—C22	118.27 (13)
N2—C3—C2	103.21 (12)	C26—C21—C5	120.23 (13)
N2—C3—H3A	111.1	C22—C21—C5	121.36 (12)
C2—C3—H3A	111.1	O2—C22—C21	121.39 (12)
N2—C3—H3B	111.1	O2—C22—C23	116.77 (12)
C2—C3—H3B	111.1	C21—C22—C23	121.83 (12)
H3A—C3—H3B	109.1	C24—C23—C22	117.96 (14)
N1—C4—C11	110.89 (11)	C24—C23—C27	123.03 (13)
N1—C4—H4A	109.5	C22—C23—C27	118.99 (13)
C11—C4—H4A	109.5	C25—C24—C23	121.28 (14)
N1—C4—H4B	109.5	C25—C24—H24	119.4
C11—C4—H4B	109.5	C23—C24—H24	119.4
H4A—C4—H4B	108.1	C24—C25—C26	119.87 (13)
N2—C5—C21	113.46 (12)	C24—C25—H25	120.1
N2—C5—H5A	108.9	C26—C25—H25	120.1
C21—C5—H5A	108.9	C25—C26—C21	120.79 (14)
N2—C5—H5B	108.9	C25—C26—H26	119.6
C21—C5—H5B	108.9	C21—C26—H26	119.6
H5A—C5—H5B	107.7	C23—C27—H27A	109.5
C16—C11—C12	118.60 (13)	C23—C27—H27B	109.5
C16—C11—C4	120.58 (13)	H27A—C27—H27B	109.5
C12—C11—C4	120.82 (13)	C23—C27—H27C	109.5
O1—C12—C13	117.43 (13)	H27A—C27—H27C	109.5
O1—C12—C11	121.33 (13)	H27B—C27—H27C	109.5
			
C4—N1—C1—N2	156.96 (11)	C12—C13—C14—C15	1.2 (2)
C2—N1—C1—N2	33.51 (15)	C17—C13—C14—C15	−176.34 (15)
C5—N2—C1—N1	115.88 (13)	C13—C14—C15—C16	−0.7 (2)
C3—N2—C1—N1	−8.89 (15)	C14—C15—C16—C11	−0.3 (2)
C1—N1—C2—C3	−44.34 (14)	C12—C11—C16—C15	0.6 (2)
C4—N1—C2—C3	−166.63 (12)	C4—C11—C16—C15	179.84 (14)
C5—N2—C3—C2	−143.72 (12)	N2—C5—C21—C26	−152.12 (12)
C1—N2—C3—C2	−18.22 (16)	N2—C5—C21—C22	32.45 (18)
N1—C2—C3—N2	38.30 (15)	C26—C21—C22—O2	−178.73 (12)
C1—N1—C4—C11	65.00 (15)	C5—C21—C22—O2	−3.2 (2)
C2—N1—C4—C11	−177.72 (11)	C26—C21—C22—C23	−0.2 (2)
C1—N2—C5—C21	74.79 (14)	C5—C21—C22—C23	175.35 (12)
C3—N2—C5—C21	−163.60 (11)	O2—C22—C23—C24	177.86 (12)
N1—C4—C11—C16	−135.44 (13)	C21—C22—C23—C24	−0.8 (2)
N1—C4—C11—C12	43.76 (17)	O2—C22—C23—C27	−0.48 (19)
C16—C11—C12—O1	178.67 (13)	C21—C22—C23—C27	−179.12 (13)
C4—C11—C12—O1	−0.5 (2)	C22—C23—C24—C25	0.9 (2)
C16—C11—C12—C13	−0.1 (2)	C27—C23—C24—C25	179.21 (14)
C4—C11—C12—C13	−179.28 (12)	C23—C24—C25—C26	−0.2 (2)
O1—C12—C13—C14	−179.60 (13)	C24—C25—C26—C21	−0.8 (2)
C11—C12—C13—C14	−0.8 (2)	C22—C21—C26—C25	1.0 (2)
O1—C12—C13—C17	−2.0 (2)	C5—C21—C26—C25	−174.60 (13)
C11—C12—C13—C17	176.75 (13)		

### Hydrogen-bond geometry (Å, º)

**Table:**

*D*—H···*A*	*D*—H	H···*A*	*D*···*A*	*D*—H···*A*
O1—H1···N1	0.91 (2)	1.90 (2)	2.7115 (16)	146 (2)
O2—H2···N2	0.98 (2)	1.70 (2)	2.6202 (16)	155 (2)
C5—H5*B*···O2^i^	0.99	2.43	3.4012 (18)	167
C27—H27*A*···O2^ii^	0.98	2.50	3.1789 (19)	126

Symmetry codes: (i) −*x*, −*y*, −*z*+1; (ii) *x*, −*y*+1/2, *z*+1/2.
